# Direct observation of electrically induced Pauli paramagnetism in single-layer graphene using ESR spectroscopy

**DOI:** 10.1038/srep34966

**Published:** 2016-10-12

**Authors:** Naohiro Fujita, Daisuke Matsumoto, Yuki Sakurai, Kenji Kawahara, Hiroki Ago, Taishi Takenobu, Kazuhiro Marumoto

**Affiliations:** 1 Division of Materials Science, University of Tsukuba, Tsukuba, Ibaraki 305-8573, Japan; 2 Global Innovation Center, Kyushu University, Fukuoka 816-8580, Japan; 3 Department of Applied Physics, Waseda University, Tokyo 169-8555, Japan; 4 Tsukuba Research Center for Interdisciplinary Materials Science (TIMS), University of Tsukuba, Ibaraki 305-8570, Japan

## Abstract

Graphene has been actively investigated as an electronic material owing to many excellent physical properties, such as high charge mobility and quantum Hall effect, due to the characteristics of a linear band structure and an ideal two-dimensional electron system. However, the correlations between the transport characteristics and the spin states of charge carriers or atomic vacancies in graphene have not yet been fully elucidated. Here, we show the spin states of single-layer graphene to clarify the correlations using electron spin resonance (ESR) spectroscopy as a function of accumulated charge density using transistor structures. Two different electrically induced ESR signals were observed. One is originated from a Fermi-degenerate two-dimensional electron system, demonstrating the first observation of electrically induced Pauli paramagnetism from a microscopic viewpoint, showing a clear contrast to no ESR observation of Pauli paramagnetism in carbon nanotubes (CNTs) due to a one-dimensional electron system. The other is originated from the electrically induced ambipolar spin vanishments due to atomic vacancies in graphene, showing a universal phenomenon for carbon materials including CNTs. The degenerate electron system with the ambipolar spin vanishments would contribute to high charge mobility due to the decrease in spin scatterings in graphene.

Graphene has attracted a great deal of attention because graphene shows excellent physical properties, such as quantum anomalous Hall effect^1^ and ballistic transport^2^,^3^. Graphene has a honeycomb structure formed with carbon atoms and is an ideal two-dimensional material^4^,^5^,^6^. Graphene has the Dirac point without a band gap in the energy dispersion relation, where electrons are regarded as Dirac particles and the effective mass is zero. As a result, the charge mobility becomes very high and the transport properties have been actively studied^7^. Also, its application to high-speed transistors in electrical engineering is expected in the future^8^,^9^,^10^.

To further understand the high mobility and to apply graphene to the high-speed transistors, the elucidation of the spin and charge-impurity states that cause charge carrier scatterings^11^,^12^,^13^,^14^ and the solution of the band gapless structure^15^,^16^,^17^,^18^,^19^ are necessary. Especially, understanding of the spin states is considered to be very important because it also leads to the applications to other areas as a new functional material. A spin-polarized density functional theory (DFT) calculation has shown the spin formation due to atomic vacancies existing in graphene, which is related to the charge carrier scatterings^20^. Such atomic vacancies have been observed by transmission electron microscope study^21^. However, the detailed studies for the spin states in graphene devices and for the correlations between the transport characteristics and the spin states have not yet been sufficiently carried out from a microscopic viewpoint. Such studies would reveal the relationships between the charge transport and the carrier scatterings, which is needed to fully utilize graphene as the electronic material.

Electron spin resonance (ESR) spectroscopy using device structures is the most effective method to study the spin states in electronic materials under device operations^22^,^23^,^24^,^25^,^26^. The ESR method has the advantages that one can directly observe the charge carriers in electronic materials and devices nondestructively, and has revealed microscopic properties, such as spin states and trap states of charge carriers in materials and devices^22^,^23^,^24^,^25^,^26^. The graphene materials have been studied by ESR^27^,^28^. However, the ESR study of the spin states in graphene devices has not yet been carried out due to the difficulties in fabricating single-layer graphene with large area and high quality.

Here, we report the ESR study of electrically induced spin states in single-layer graphene that are carried out using driven transistors fabricated with large-area high-quality graphene. To carry out the ESR measurements sensitively, we use an ion gel which is capable of forming high charge-density states in graphene^29^,^30^,^31^. From the measurements of ESR and transistor characteristics, we have observed two types of the ESR signals with different gate-voltage dependences. One is originated from the Fermi-degenerate two-dimensional electron system, which is the first observation of electrically induced Pauli paramagnetism in graphene, and is directly related to the transport characteristics. The other is originated from electrically induced ambipolar spin vanishments due to atomic vacancies in graphene, as observed in carbon nanotubes (CNTs)^26^. These results provide insights into the relationships between the excellent charge-transport properties and the spin scatterings mechanisms in graphene.

To attain high signal-to-noise (S/N) ratio of the ESR signal by increasing the active area of the device, we utilized a rectangular graphene-transistor structure (3 mm × 30 mm) in an ESR sample tube with an inner diameter of 3.5 mm. Figure. 1a shows a schematic of the device structure. The formation of single-layer graphene was confirmed with the Raman spectra as shown Fig. S1 in Supplementary Information. We were able to achieve a low-voltage transistor operation by applying an ion-gel insulator to the device structure owing to the formation of the electric double layers at the interface between graphene and the insulator^29^,^30^,^31^. For the source (S), drain (D), and gate (G) electrodes, Ni (1 nm)/Au (30 nm) were vapour-deposited unless otherwise stated. The details of the fabrication methods of the single-layer graphene samples and the transistor structures are described in Methods.

The spin states of single-layer graphene are clearly reflected in the ESR signals. We show the ESR signals of graphene transistors under a wide gate-voltage (*V*_G_) region to present the microscopic investigations of the spin states due to the Fermi-degenerate two-dimensional electron system and those due to the atomic vacancies in graphene. Figure 1b shows the ESR spectra of the graphene transistor when applying negative and positive *V*_G_. In the negative *V*_G_ region, the ESR signal intensity increased with increasing the absolute value of the *V*_G_. The parameters of the ESR signal at *V*_G_ = −1.5 V were obtained as the *g* factor of *g* = 2.0036 and the peak-to-peak ESR linewidth (*ΔH*_pp_) of 1.45 mT. In contrast, the ESR spectrum and the *V*_G_ dependence dramatically changed under the positive *V*_G_ region, as shown in Fig. 1b. The ESR signal intensity increased as the *V*_G_ increased and showed a maximum at *V*_G_ = 0.8 V, and then decreased when the *V*_G_ increased from *V*_G_ = 0.8 V. The ESR parameters at *V*_G_ = 0.8 V were obtained as *g* = 2.0033 and *ΔH*_pp_ = 0.74 mT. As discussed later in detail, the signals at *V*_G_ = −1.5 and 0.8 V are ascribed to the charge carriers and the atomic vacancies in graphene, respectively.

The correlation between the transport characteristics and the observed ESR signals were examined by measuring the *V*_G_ dependence of the ESR intensity and the sheet conductivity (*σ*_2D_) of the graphene transistor. To present the ESR intensity, we evaluated the spin susceptibility (*χ*) from double integral value of the ESR spectrum, considering the active area of the graphene transistor. The *σ*_2D_ value showed a minimum at *V*_G_ = 0.8 V or at *V*_G_ = 0.6 V when the source and drain electrodes of Ni/Au or Au were used, respectively. The observation of the *σ*_2D_ minimum indicates an existence of the charge neutral point, namely, the Dirac point in the graphene transistors. Such behavior is consistent with that reported for single-layer graphene transistors^32^,^33^. We define the *V*_G_ at the charge neutral point as *V*_CNP_. Figure 1c shows the dependence of the *χ* and the *σ*_2D_ on *V*_G_ − *V*_CNP_. The *χ* value shows a maximum at *V*_G_* = V*_CNP_ where the *σ*_2D_ shows the minimum. The increase in the *χ* has been observed for *V*_G_ − *V*_CNP_ < −1 V. It is worth noting that the maximum of the *χ* at *V*_G_ = *V*_CNP_ with the drain-current minimum is fully consistent with that observed for the CNT transistors^26^.

The ESR signal due to the charge carriers should have a correlation with the transport characteristics. To demonstrate the correlation, we discuss the relationship between the spin susceptibility due to the charge carriers and the *σ*_2D_ of the graphene transistor. We extracted the Lorentzian component from the ESR spectrum of the graphene transistor because mobile charge carries show an ESR signal with the Lorentzian lineshape. We define the spin susceptibility due to the charge carriers as *χ*_L_, which was evaluated from double integral value of the ESR spectrum with the Lorentzian lineshape. The *χ*_L_ at different *V*_G_ was evaluated by performing the ESR spectrum simulation using Lorentzian and Gaussian functions. Figure 2a shows the fitting analysis for the ESR spectrum at *V*_G_ = −1.5 V (or *V*_G_ − *V*_CNP_ = −2.3 V), where most of the signal component is found to be described by the Lorentzian function. In contrast, the fitting analysis for the ESR spectrum at *V*_G_ = 0.8 V (or *V*_G_ − *V*_CNP_ = 0 V) exhibits a different result, where the signal component is found to be described practically by the Gaussian function, as shown in Fig. 2b. The vertical magnitude of the Lorentzian component is very small compared with that of the Gaussian component in the ESR spectrum at *V*_G_ = 0.8 V because the *χ*_L_ is proportional to the square of the *ΔH*_pp_ and the *ΔH*_pp_ of the Lorentzian component is considerably broader than that of the Gaussian component. Figure 2c,d show the dependence of the *σ*_2D_ and the *χ*_L_ of the graphene transistor on *V*_G_ − *V*_CNP_, respectively. It is worth noting that the *χ*_L_ value shows a minimum at *V*_G_ = *V*_CNP_, which is consistent with the minimum of the *σ*_2D_. The increases in the *χ*_L_ for *V*_G_ < *V*_CNP_ and *V*_G_ > *V*_CNP_ have been observed, which are ascribed to the accumulation of holes and electrons, respectively. Therefore, the clear correlation between the *χ*_L_ and the *σ*_2D_ demonstrates that the ESR signal with the Lorentzian lineshape is originated from the charge carriers in graphene. Schematics of the energy diagram and the spin states in the graphene transistor are shown in Fig. 3. The *χ*_L_ intensity reflects the density of states at the Fermi surface in graphene shown in Fig. 3a,c. The result exhibits the first ESR observation due to electrically induced ambipolar carriers in the Fermi-degenerate electron system in graphene.

The Fermi-degenerate electron system is known to show no temperature dependence for the spin susceptibility *χ*, which is called as Pauli paramagnetism. Thus, it is interesting to discuss the temperature dependence of the *χ*. The previous studies have reported the temperature dependence of the ESR intensity of the graphene samples^27^,^28^. Although these studies showed that the *χ* of the graphene samples followed the Curie law or the Curie-Weiss law^27^,^28^, the Pauli paramagnetism has not yet been observed. Thus, we carried out the measurements for the temperature dependence of the *χ* using the graphene transistor to observe the Pauli paramagnetism in graphene. Figure 4a shows the temperature dependence of the *χ* of the graphene transistor. The *χ* was evaluated from the ESR signal at *V*_G_ = −1.5 V (or *V*_G_ − *V*_CNP_ = −2.3 V) for the hole accumulation state by double integrating the ESR spectrum. As shown in Fig. 4a, the *χ* hardly depends on the temperature, which means the Pauli paramagnetism, not the Curie paramagnetism shown by dashed line in Fig. 4a. The Pauli paramagnetism in graphene has been observed by ESR measurements for the first time. Therefore, we demonstrate the formation of the Fermi-degenerate two-dimensional electron system in graphene from a microscopic viewpoint.

The states of the electron system is also reflected in the temperature dependence of the ESR linewidth. Figure 4b shows the temperature dependence of the full width at half maximum of the ESR signal (*ΔH*_1/2_), which was obtained by integrating the ESR spectrum. As shown in Fig. 4b, the *ΔH*_1/2_ increases with increasing temperature. This result can be explained by the Elliott mechanism observed for Fermi-degenerate electron systems, where the spin-lattice relaxation time (*T*_1_) is shortened with increasing temperature and the ESR linewidth is proportional to 1/*T*_1_, that is, *ΔH*_1/2_ ∝ 1/*T*_1_ (ref. 34). Therefore, the behavior of the temperature dependence of the ESR linewidth also supports the formation of the Fermi-degenerate two-dimensional electron system in graphene.

We now turn to a discussion of the ESR study of the atomic vacancies in graphene. In the theoretical study of graphene, the existence of atomic vacancies and the corresponding localized spin states have been discussed^20^. Thus, it is interesting to discuss the existence of the atomic vacancies in graphene by observing the spin states using the ESR method. To discuss the ESR signal due to the atomic vacancies in graphene, we extracted the Gaussian component from the ESR spectrum of the graphene transistor because localized spins show an ESR signal with the Gaussian lineshape. We define the spin susceptibility due to the atomic vacancies as *χ*_G_, which was evaluated from the double integral value of the ESR spectrum with the Gaussian lineshape. In other words, *χ*_G_ = *χ* − *χ*_L_. Figure 2e shows the dependence of the *χ*_G_ on *V*_G_ − *V*_CNP_. The *χ*_G_ shows a maximum at *V*_G_ = *V*_CNP_ and decreases for *V*_G_ < *V*_CNP_ and *V*_G_ > *V*_CNP_. Notably, the *χ*_G_ decreases and the *σ*_2D_ inversely increases as the *V*_G_ varies from *V*_CNP_. That is, this result shows an inverse correlation between the *χ*_G_ and the *σ*_2D_. The inverse correlation is in contrast to the direct correlation between the *χ*_L_ and the *σ*_2D_ shown in Fig. 2c,d. As discussed in the ESR study of the CNT transistors^26^, the inverse correlation demonstrates electrically induced ambipolar spin vanishments due to the atomic vacancies in graphene, as explained below.

The electrically induced ambipolar spin vanishments have been reported in the ESR study of the CNT transistors, where the number of spins (*N*_spin_) due to the atomic vacancies shows a maximum at a charge neutral point (*V*_G_ = *V*_CNP_) with a drain-current minimum^26^. Thus, it is interesting to compare the *N*_spin_ due to the atomic vacancies in the graphene transistors with that of the CNT transistors to present the universality of the electrically induced ambipolar spin vanishment in carbon materials. We evaluated the *N*_spin_ in the graphene transistors from the double integral value of the ESR spectrum with the Gaussian lineshape. The *N*_spin_ is related to the *χ*_G_ as 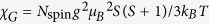 where *μ*_*B*_ is the Bohr magneton, *S* is a spin quantum number due to an atomic vacancy (*S* = 1/2), *k*_*B*_ is the Boltzmann constant, and *T* is the temperature. Figure 5a,b show the dependence of the *N*_spin_ of the graphene transistor and the CNT transistors on *V*_G_ − *V*_CNP_, respectively. The data for the CNT transistor are taken from the literature^26^. The *N*_spin_ similarly has the maximum at *V*_G_ = *V*_CNP_ and decreases for *V*_G_ < *V*_CNP_ and *V*_G_ > *V*_CNP_ in the ideal two-dimensional material single-layer graphene and in the one-dimensional material CNTs, respectively. Therefore, the electrically induced ambipolar spin vanishment seems a universal phenomenon for carbon materials with honeycomb structures formed with carbon atoms regardless of the dimensionality of the electron systems.

The electrically induced ambipolar spin vanishments are schematically explained in Fig. 3a–f. The graphene transistor shows the charge neutral point at *V*_G_ = *V*_CNP_ where the *N*_spin_ (or the *χ*_G_) due to non-bonding orbitals (NBOs) at the atomic vacancies in graphene shows the maximum value (see Fig. 3b,e). In the region for *V*_G_ > *V*_CNP_, electrically induced spin vanishments are explained by spin pairings between the spins of electrically induced electrons by the *V*_G_ and the spins due to the NBOs at the atomic vacancies, which decreases the *N*_spin_ due to the cancelation of each spin (see Fig. 3a,d). This result indicates the existence of the antiferromagnetic interactions between these spins of the electrically induced electrons and the atomic vacancies in graphene. In the region for *V*_G_ < *V*_CNP_, electrically induced spin vanishments are explained by the discharges of the vacancies’ spins due to hole doping by the *V*_G_ (see Fig. 3c,f). That is, the spins of the atomic vacancies are released. We here comment on the difference between the dependence of the *N*_spin_ on the *V*_G_ of the graphene transistor and CNT transistor shown in Fig. 5a,b. While the former steeply changes at *V*_G_ − *V*_CNP_ < −0.4 V and *V*_G_ − *V*_CNP_ > 0.2 V, the latter changes almost linearly to the *V*_G_. The almost linear change may indicate a distribution of charge density (or ion liquid) in the CNT thin film due to a thick film thickness of CNT (approximately 300 nm)^26^. The ESR study of the ion-gel-gated polymer transistors has indicated a gradient in charge density normal to the transistor channel, with higher charge density near the polymer/ion-gel interface^24^. Such charge-density distribution in the CNT thin film may gradually cause the electrically induced spin vanishments with respect to the variation of *V*_G_, resulting in the almost linear change in the *N*_spin_ . In the case of graphene, such charge-density distribution is not basically expected due to a monolayer film thickness.

In contrast to the similar observation of the electrically induced ambipolar spin vanishments in the graphene and CNT transistors, the ESR signal due to charge carries was unable to be observed in the CNT transistors^26^. The reason for the non-observation of the ESR signal due to the charge carriers in CNTs has been explained by the formation of an one-dimensional electron system, that is, Tomonaga-Luttinger-liquid (TLL) states^26^,^35^,^36^. On the basis of the framework of TLL theory with spin-symmetry breaking and electron-electron interaction in the one-dimensional electron system, the previous studies have showed that the ESR linewidth due to TLL states becomes extremely broad, on the order of 100 mT, even at low temperatures such as 4 K, and further broadening of the ESR linewidth at higher temperatures^26^,^35^,^36^. Such extremely broadening of the ESR linewidth makes the ESR signal undetectable. Thus, the dimensionality of the electron systems is an important factor for observing the ESR signal due to the charge carries in carbon materials. The clear observation of the ESR signal due to the charge carries in graphene confirms the two-dimensional electron system from a microscopic viewpoint.

Our result demonstrates the observation of the ESR signal due to the charge carriers and the electrically induced Pauli paramagnetism in the Fermi-degenerate two-dimensional electron system in graphene. The result is interesting due to the first direct ESR observation of the electrically induced Pauli paramagnetism for organic, inorganic, and carbon materials. The observation also demonstrates the existence of the Fermi surface of the two-dimensional material graphene from a microscopic viewpoint. Moreover, the electrically induced ambipolar spin vanishments due to the atomic vacancies in graphene are observed, which indicates that the spin vanishment is a universal phenomenon in carbon materials including carbon nanotubes regardless of the dimensionality of the electron systems. The spins due to the atomic vacancies vanish owing to the antiferromagnetic interactions with electrically induced electrons or the discharges of the vacancies’ spins due to hole doping, which would decrease the carriers’ spin scatterings due to the atomic vacancies. Therefore, the Fermi-degenerate electron system with the electrically induced ambipolar spin vanishments would contribute to high charge mobility in graphene. Our approach using the electrically induced ESR technique is the most appropriate method for investigating the spin states and their correlations with the transport characteristics in electronic materials from a microscopic viewpoint because the method can directly observe the correlations between the spin states and the transport characteristic. Applying the method to other various electronic materials would give new understanding of the microscopic properties which are also useful for improving the device performance.

## Methods

### Fabrication of single-layer graphene transistors

We synthesized large-area single-layer graphene with a chemical vapor deposition (CVD) method^37^,^38^. The fabrication processes are as follows^38^. A 1000-nm-thick Cu(111) film was deposited on *c*-plane sapphire [*α*-Al_2_O_3_(0001)] by magnetron sputtering at high temperature. The Cu/sapphire substrate was placed inside a quartz tube (inner diameter 26 mm) and then annealed at 1000°C in the flow of H_2_ (2.5% in Ar) gases for 40 min. After elevating the temperature to 1075 °C, CH_4_ gas (10 ppm) was introduced for 90 min to grow graphene. High purity gases were used for this experiment (>99.999% purity for all the gases). Confocal Raman (Tokyo Instruments Nanofinder30 with 532 nm excitation) measurements were performed for the graphene transferred on a quartz substrate by a poly(methyl methacrylate) (PMMA) mediated transfer technique. The obtained Raman spectra are consistent with that previously reported for single-layer graphene^39^, which confirms the fabrication of single-layer graphene on the quartz substrate, as shown Fig. S1 in Supplementary Information.

The transistors were fabricated using two types of nonmagnetic substrates; one was a polyethylene terephthalate (PET) film with dimensions of 30 mm × 3 mm × 100 μm (Mitsubishi Polyester Film, Inc.), and the other was a quartz glass with dimensions of 30 mm × 3 mm × 1 mm (IIYAMA PRECISION GLASS Co, Ltd.). Gate electrodes of Ni (1 nm) and Au (30 nm) were vapour-deposited on the PET substrate using an ULVAC VPC-260F vacuum evaporation system. Ion-gel solutions consisted of an ionic liquid, 1-ethyl-3-methylimidazolium bis(trifluoromethylsulfonyl)imide ([EMIM][TFSI]) (36 wt%) (Ionic Liquids Technologies, Inc.), a gelator ABA-type triblock copolymer poly(styrene-*b*-methylmethacylate-*b*-styrene) (PS-PMMA-PS) (3 wt%) (Polymer Source, Inc.), and a solvent ethyl acetate (61 wt%) (Wako Pure Chemical Industries, Ltd.); the mixture was stirred for over one and half day, drop-casted on the gate electrode and then thermally annealed at 70 °C under vacuum for over one and half day. The ion-gel insulator shows large electric double layer (EDL) capacitance and high ionic conductivity. The EDL capacitance is generally very large (~10–100 μF cm^−2^), leading to significant charge accumulation with low voltage and high on/off current ratios. The source and drain electrodes of Ni (1 nm)/Au (30 nm) or Au (30 nm) which had a channel length of 1.0 mm and a channel width of approximately 25 mm were fabricated with a vapour-deposition method on the quartz substrate. Finally, the PET substrate was placed on the quartz substrate, completing the transistor fabrication. The fabricated transistor was sealed into an ESR sample tube under a nitrogen glove-box atmosphere (O_2_ < 0.2 ppm, H_2_O < 0.5 ppm) after the transistor was connected to wires by Ag paste.

### ESR and transfer characteristic measurements

The ESR measurements were performed with a JEOL RESONANCE JES-FA200 X-band ESR spectrometer and a Keithley 2612A source meter. The ESR signals were measured as a function of *V*_G_ by averaging the ESR spectrum over typically 10–30 min. The *g* factor and the linewidth of the ESR signals were calibrated using a standard Mn^2+^ marker sample. The peak-to-peak ESR linewidth, Δ*H*_pp_, was evaluated as the difference between the two magnetic fields at a peak and valley in the ESR spectrum. The ESR linewidth, Δ*H*_1/2_, was evaluated from the full width at half maximum (FWHM) of the first integrated ESR spectrum. The spin susceptibility (*χ*, *χ*_L_, and *χ*_G_) was evaluated by integrating the ESR spectrum twice and comparing it with that of the Mn^2+^ marker sample. The number of spins, *N*_spin_, was evaluated by integrating the ESR spectrum twice and by comparing it with that of the Mn^2+^ marker sample. The absolute value of the number of spins of the Mn^2+^ marker sample was calculated using a solution (220 μL) of 4-hydroxy-2,2,6,6-tetramethylpiperidin-1-oxyl (TEMPOL) as a standard. The calibration of the *g* factor was performed using a software program from the JEOL RESONANCE ESR system considering high second-order correction to the effective resonance field. Its correctness was also confirmed using 2,2-diphenyl-1-picrylhydrazyl (DPPH) as another standard sample.

## Additional Information

**How to cite this article**: Fujita, N. *et al.* Direct observation of electrically induced Pauli paramagnetism in single-layer graphene using ESR spectroscopy. *Sci. Rep.* **6**, 34966; doi: 10.1038/srep34966 (2016).

## Supplementary Material

Supplementary Information

## Figures and Tables

**Figure 1:**
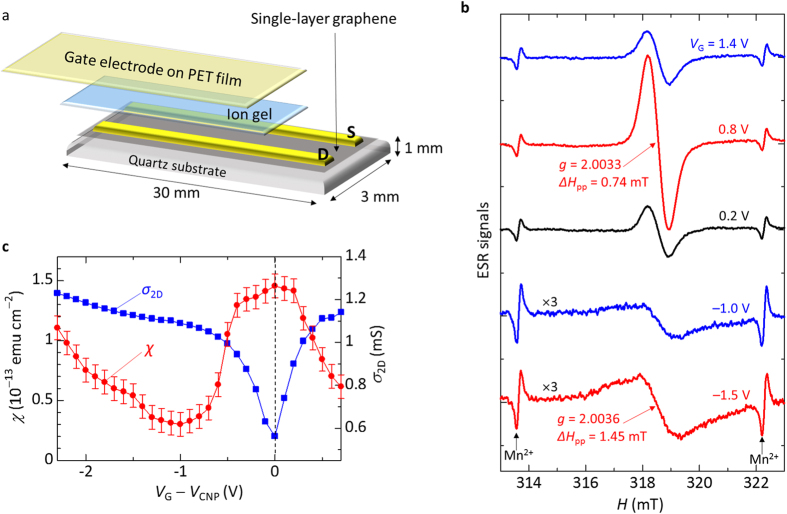
Schematics of a graphene transistor and the ESR and device characteristics. (**a**) Schematic of the device structure of a graphene transistor used in this study. (**b**) ESR spectra of the graphene transistor at positive and negative *V*_G_, where *V*_D_ = 0.1 V at the external magnetic field *H* perpendicular to the substrate (*H*_⊥_) at 300 K. (**c**) Dependence of the spin susceptibility, *χ*, and the sheet conductivity, *σ*_2D_, of the graphene transistor on *V*_G_ − *V*_CNP_, where *V*_D_ = 0.1 V at *H*_⊥_ at 300 K. The *V*_CNP_ is defined as the *V*_G_ at a charge neutral point in the graphene transistor.

**Figure 2:**
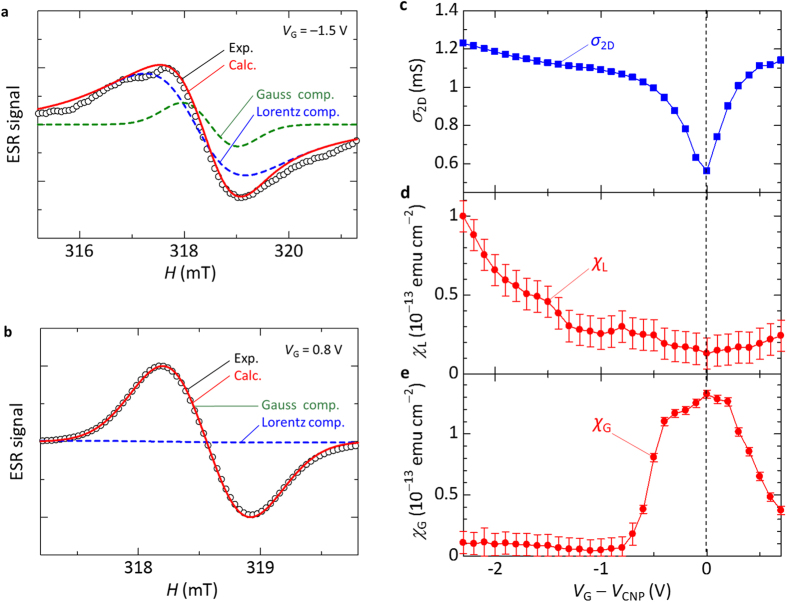
Spin susceptibility due to the charge carries (Lorentzian component) and the atomic vacancies (Gaussian component) of the graphene transistor. (**a,b**) Fitting analysis for the ESR spectrum of the graphene transistor at *V*_G_ = −1.5 V (**a**) and at *V*_G_ = 0.8 V (**b**), respectively. Black circles represent the experimental results. Blue and green dashed lines represent the Lorentzian and Gaussian components, respectively, and the red solid line represents the sum of the Lorentzian and Gaussian components. (**c**–**e**), Dependence of the *σ*_2D_ (**c**), the spin susceptibility due to the charge carries, *χ*_L_, (Lorentzian component) (**d**), and the spin susceptibility due to the atomic vacancies, *χ*_G_, (Gaussian component) (**e**) of the graphene transistor on *V*_G_ − *V*_CNP_, where *V*_D_ = 0.1 V at *H*_⊥_ at 300 K.

**Figure 3:**
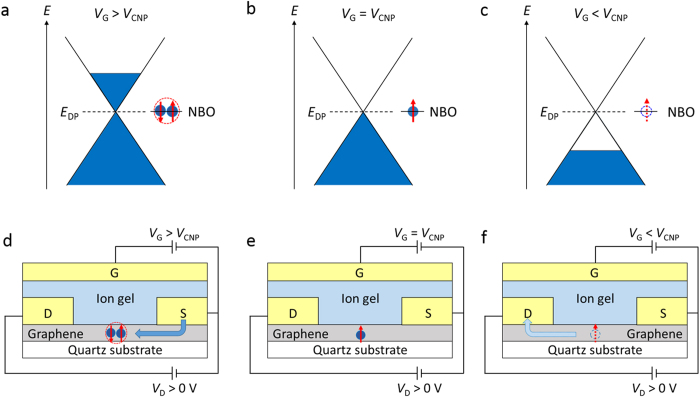
Energy diagram and spin states in the graphene transistor. (**a**–**c**) Schematics of the energy diagram and non-bonding orbital (NBO) of graphene with spin states for *V*_G_ > *V*_CNP_ (**a**), at *V*_G_ = *V*_CNP_ (**b**), and for *V*_G_ < *V*_CNP_ (**c**). The energy level of the NBO is located at the energy of the Dirac point (*E*_DP_). (**d**–**f**) Schematics of the spin states in the graphene transistor for *V*_G_ > *V*_CNP_ (**d**), at *V*_G_ = *V*_CNP_ (**e**), and for *V*_G_ < *V*_CNP_ (**f**).

**Figure 4:**
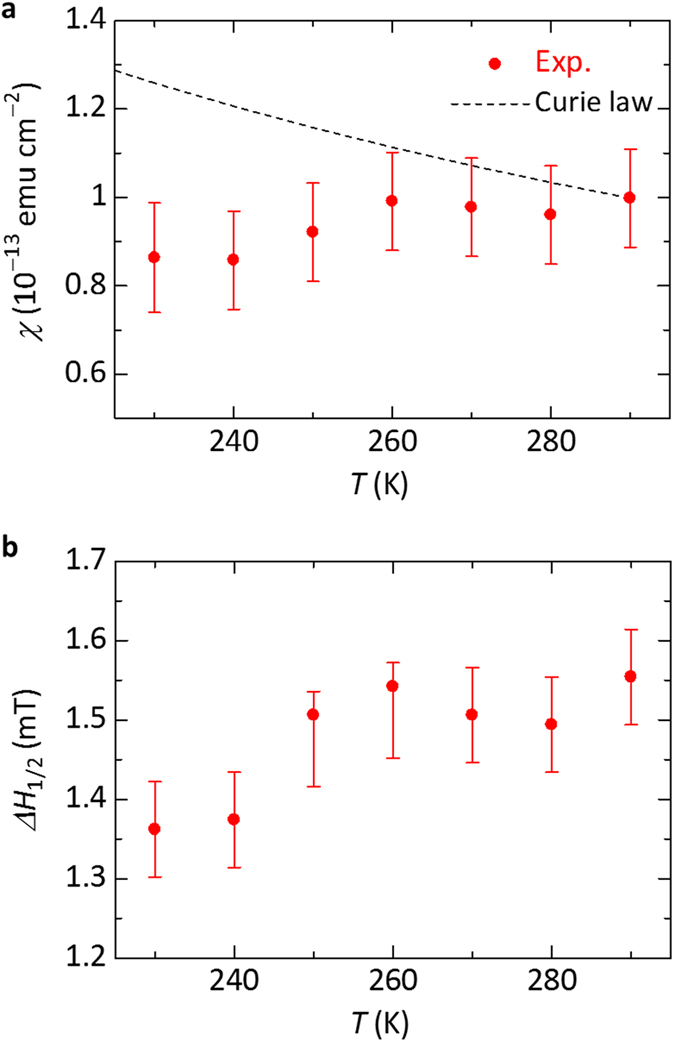
Temperature dependence of the ESR parameters of the graphene transistor. (**a**) Temperature dependence of the *χ* of the graphene transistor, where *V*_G_ = −1.5 V and *V*_D_ = 0.1 V at *H*_⊥_. The dashed line represents the temperature dependence of the susceptibility following the Curie law. (**b**) Temperature dependence of the *ΔH*_1/2_ of the graphene transistor, where *V*_G_ = −1.5 V and *V*_D_ = 0.1 V at *H*_⊥_.

**Figure 5:**
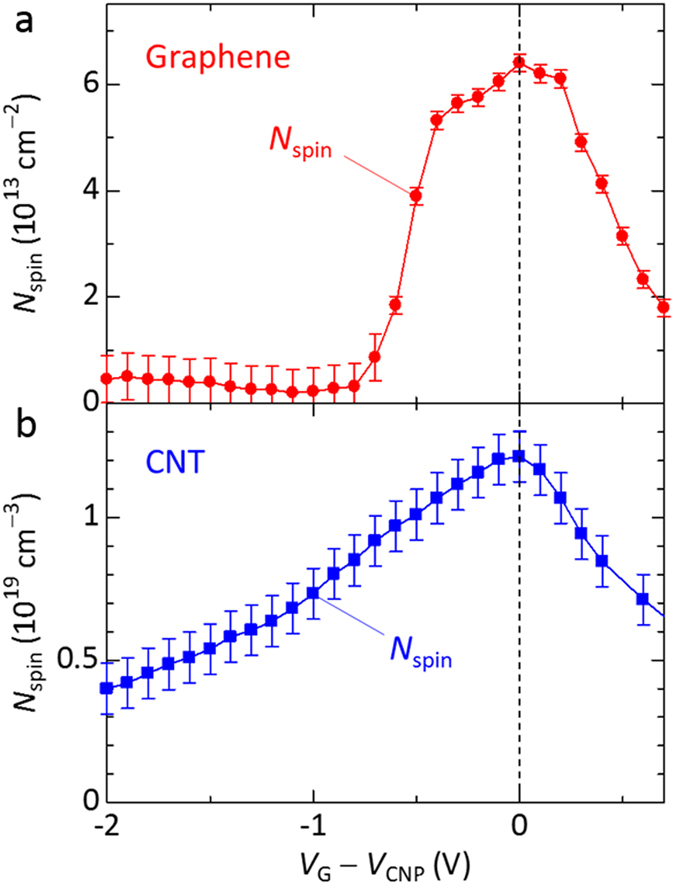
Electrically induced ambipolar spin vanishments due to the atomic vacancies. (**a**,**b**) Dependence of the number of spins (*N*_spin_) due to the atomic vacancies in the graphene transistor (**a**) and in the CNT transistor (**b**) on *V*_G_ − *V*_CNP_. The data for the CNT transistor are taken from the literature^26^.
