# Supplementary material for: Direct observation of electrically induced Pauli paramagnetism in single-layer graphene using ESR spectroscopy
Source: Sci Rep. 2016 Oct 12;6:34966. doi: 10.1038/srep34966 (PMC5059738; doi:10.1038/srep34966)
Supplement: Supplementary Information [file srep34966-s1.pdf]

Supplementary Information for

Direct observation of electrically induced Pauli paramagnetism of single-layer graphene using ESR spectroscopy

Naohiro Fujita, Daisuke Matsumoto, Yuki Sakurai, Kenji Kawahara, Hiroki Ago, Taishi Takenobu and Kazuhiro Marumoto*

*To whom correspondence should be addressed; E-mail:marumoto@ims.tsukuba.ac.jp

S1. Raman spectroscopy of single-layer graphene

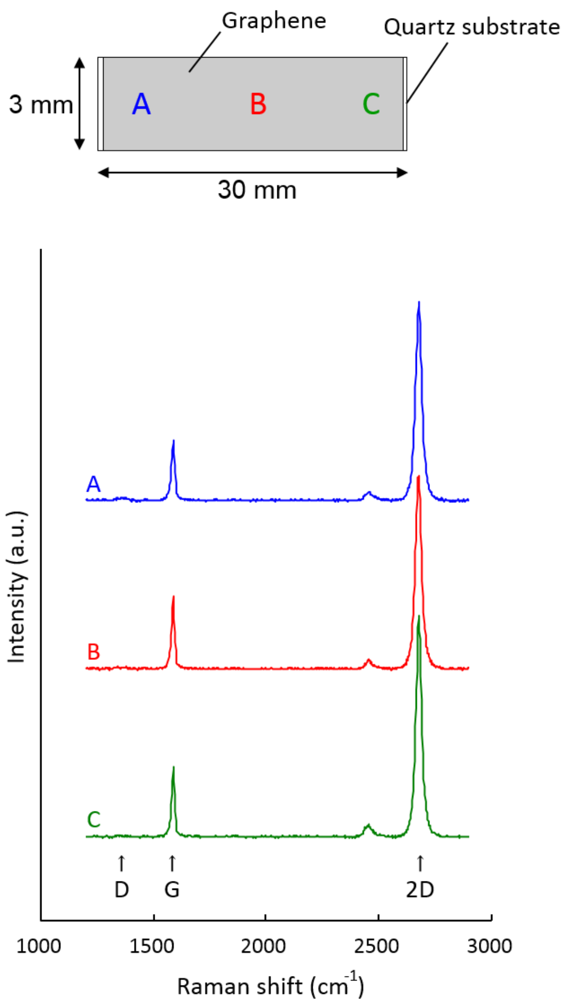

Figure S1 | Raman spectra of single-layer graphene. The Raman spectra of the single-layer graphene sample on a quartz substrate. Signals A, B, and C were measured at different positions A, B, and C on the substrate, respectively.

The obtained Raman spectra are consistent with that previously reported for single-layer graphene[1], which confirms the synthesis of single-layer graphene on the quartz substrate. The Raman spectra measured at the different positions A, B, and C are consistent with each other, which confirms that single-layer graphene is uniformly formed on the quartz substrate.

Reference

1. Ferrari, A. C. *et al*. Raman Spectrum of Graphene and Graphene Layers. *Phys. Rev. Lett* **97**, 187401 (2006).
